# Association of eGFR-Related Loci Identified by GWAS with Incident CKD and ESRD

**DOI:** 10.1371/journal.pgen.1002292

**Published:** 2011-09-29

**Authors:** Carsten A. Böger, Mathias Gorski, Man Li, Michael M. Hoffmann, Chunmei Huang, Qiong Yang, Alexander Teumer, Vera Krane, Conall M. O'Seaghdha, Zoltán Kutalik, H.-Erich Wichmann, Thomas Haak, Eva Boes, Stefan Coassin, Josef Coresh, Barbara Kollerits, Margot Haun, Bernhard Paulweber, Anna Köttgen, Guo Li, Michael G. Shlipak, Neil Powe, Shih-Jen Hwang, Abbas Dehghan, Fernando Rivadeneira, André Uitterlinden, Albert Hofman, Jacques S. Beckmann, Bernhard K. Krämer, Jacqueline Witteman, Murielle Bochud, David Siscovick, Rainer Rettig, Florian Kronenberg, Christoph Wanner, Ravi I. Thadhani, Iris M. Heid, Caroline S. Fox, W. H. Kao, The CKDGen Consortium

**Affiliations:** 1 Department of Internal Medicine II, University Hospital Regensburg, Regensburg, Germany; 2 Department of Epidemiology and Preventive Medicine, University Hospital Regensburg, Regensburg, Germany; 3 Institute of Epidemiology I, Helmholtz Zentrum München, German Research Center for Environmental Health, Neuherberg, Germany; 4 Department of Epidemiology, Johns Hopkins Bloomberg School of Public Health, Baltimore, Maryland, United States of America; 5 Clinical Chemistry, University Medical Center Freiburg, University of Freiburg, Freiburg, Germany; 6 Division of Nephrology, Massachusetts General Hospital, Boston, Massachusetts, United States of America; 7 Department of Biostatistics, Boston University School of Public Health, Boston, Massachusetts, United States of America; 8 Interfaculty Institute for Genetics and Functional Genomics, University of Greifswald, Greifswald, Germany; 9 University of Würzburg, Department of Medicine 1, Division of Nephrology, University Hospital Würzburg, Würzburg, Germany; 10 Division of Nephrology, Brigham and Women's Hospital and Harvard Medical School, Boston, Massachusetts, United States of America; 11 NHLBI's Framingham Heart Study and the Center for Population Studies, Framingham, Massachusetts, United States of America; 12 Department of Medical Genetics, University of Lausanne, Lausanne, Switzerland; 13 Swiss Institute of Bioinformatics, Lausanne, Switzerland; 14 Institute of Medical Informatics, Biometry, and Epidemiology, Ludwig-Maximilians-Universität, Munich, Germany; 15 Klinikum Großhadern, Munich, Germany; 16 Diabetes Klinik Bad Mergentheim, Bad Mergentheim, Germany; 17 Innsbruck Medical University, Division of Genetic Epidemiology, Innsbruck, Austria; 18 Department of Epidemiology and Welch Center for Prevention, Epidemiology, and Clinical Research, Johns Hopkins Bloomberg School of Public Health, Baltimore, Maryland, United States of America; 19 First Department of Internal Medicine, Paracelsus Medical University, Salzburg, Austria; 20 Renal Division, University Hospital of Freiburg, Freiburg, Germany; 21 Department of Medicine, University of Washington, Seattle, Washington, United States of America; 22 Division of General Internal Medicine, San Francisco VA Medical Center, San Francisco, California, United States of America; 23 Department of Medicine, Epidemiology, and Biostatistics, University of California San Francisco, San Francisco, California, United States of America; 24 Department of Medicine, San Francisco General Hospital and University of California San Francisco, San Francisco, California, United States of America; 25 Department of Epidemiology, Erasmus Medical Center, Rotterdam, The Netherlands; 26 Member of Netherlands Consortium for Healthy Aging (NCHA), Netherlands Genomics Initiative (NGI), Leiden, The Netherlands; 27 Department of Internal Medicine, Erasmus Medical Center, Rotterdam, The Netherlands; 28 Service of Medical Genetics, Centre Hospitalier Universitaire Vaudois and Department of Medical Genetics, University of Lausanne, Lausanne, Switzerland; 29 University Medical Centre Mannheim, 5th Department of Medicine, Mannheim, Germany; 30 University Institute of Social and Preventive Medicine, Centre Hospitalier Universitaire Vaudois and University of Lausanne, Lausanne, Switzerland; 31 Cardiovascular Health Research Unit, Departments of Epidemiology and Medicine, University of Washington, Seattle, Washington, United States of America; 32 Institute of Physiology, University of Greifswald, Greifswald, Germany; 33 Division of Endocrinology, Brigham and Women's Hospital and Harvard Medical School, Boston, Massachusetts, United States of America; Stanford University Medical Center, United States of America

## Abstract

Family studies suggest a genetic component to the etiology of chronic kidney disease (CKD) and end stage renal disease (ESRD). Previously, we identified 16 loci for eGFR in genome-wide association studies, but the associations of these single nucleotide polymorphisms (SNPs) for incident CKD or ESRD are unknown. We thus investigated the association of these loci with incident CKD in 26,308 individuals of European ancestry free of CKD at baseline drawn from eight population-based cohorts followed for a median of 7.2 years (including 2,122 incident CKD cases defined as eGFR <60ml/min/1.73m^2^ at follow-up) and with ESRD in four case-control studies in subjects of European ancestry (3,775 cases, 4,577 controls). SNPs at 11 of the 16 loci (*UMOD, PRKAG2, ANXA9, DAB2, SHROOM3, DACH1, STC1, SLC34A1, ALMS1/NAT8, UBE2Q2*, and *GCKR)* were associated with incident CKD; p-values ranged from p = 4.1e-9 in *UMOD* to p = 0.03 in *GCKR*. After adjusting for baseline eGFR, six of these loci remained significantly associated with incident CKD (*UMOD, PRKAG2, ANXA9, DAB2, DACH1,* and *STC1*). SNPs in *UMOD* (OR = 0.92, p = 0.04) and *GCKR* (OR = 0.93, p = 0.03) were nominally associated with ESRD. In summary, the majority of eGFR-related loci are either associated or show a strong trend towards association with incident CKD, but have modest associations with ESRD in individuals of European descent. Additional work is required to characterize the association of genetic determinants of CKD and ESRD at different stages of disease progression.

## Author Summary

Chronic kidney disease (CKD) affects about 6%–11% of the general population, and progression to end stage renal disease (ESRD) has a significant public health impact. Family studies suggest that the risk for CKD and ESRD is heritable. Unraveling the genetic underpinning of risk for these diseases may lead to the identification of novel mechanisms and thus diagnostic and therapeutic tools. We have previously identified 16 genetic markers in association with kidney function and prevalent CKD in general population studies. However, little is known about the relevance of these SNPs to the initial development of CKD or to ESRD risk. Therefore, we have now analyzed the association of these markers with the initiation of CKD in more than 26,000 individuals from the general population using serial estimations of kidney function, and with ESRD in four case-control studies in subjects of European ancestry (3,775 cases, 4,577 controls). We show that many of the 16 markers are also associated or show a strong trend towards association with initiation of CKD, while only 2 markers are nominally associated with ESRD. Further work is required to characterize the association of genetic determinants of different stages of CKD progression.

## Introduction

Chronic kidney disease (CKD) and end stage renal disease (ESRD) are associated with significant cardiovascular morbidity and mortality, with substantial economic burden [1]–[4]. Diabetes and hypertension are the primary risk factors for CKD and ESRD [5]–[8] but do not fully account for CKD and ESRD risk [9]–[11]. Studies indicate familial aggregation of ESRD [12]. In African Americans, high risk common variants in the *MYH9/APOL1* locus account for much of the excess genetic risk for non-diabetic ESRD compared to their counterparts of European descent. In contrast, comparable genetic risk loci of severe renal phenotypes have not been identified in individuals of European ancestry [13]–[15].

Recently, 16 genetic risk loci associated with estimated glomerular filtration rate (eGFR) and prevalent CKD were identified and replicated by genome wide association studies (GWAS) in about 70,000 individuals of European ancestry in the CKDGen consortium [16], [17]. Two of these loci were also identified by an independent consortium [18]. However, these studies focused on eGFR and prevalent CKD (defined as eGFR <60 ml/min/1.73m^2^) at one time point, which encompasses the entire spectrum of CKD, and does not does not address the question of whether these genetic factors are involved in the initiation of CKD or in the progression to ESRD, the most advanced stage of CKD. We thus sought to analyze the association of the previously identified 16 eGFR-associated loci with the development of CKD and with ESRD in a total of over 34,000 individuals of European descent.

## Results

### Association of SNPs with Incident CKD

Overall, 26,308 individuals of European descent, from eight population-based prospective studies, who were free of CKD at baseline were included in the incident CKD analysis (Table 1). At baseline, mean age ranged from 40.5 to 71.7 years. After a median follow-up of 7.2 years, 2122 participants developed incident CKD.

**Table 1:** Cohort characteristics of the incident CKD analysis (n = 26,308).

	n	Incident CKD cases, % (n)	Mean Age (yrs)	Women (%)	DM (%)	HTN (%)	eGFR (baseline)	eGFR (follow-up)	Duration between baseline and follow-up (Years)
ARIC	8735	8.3 (728)	54.2	52.7	8.4	26.3	90.8	82.0	7.6
CHS	2389	12.3 (295)	71.7	60.8	11.4	33.2	86.2	83.9	5.9
CoLaus	1842	4.1 (75)	53.4	54.2	5.7	34.0	93.1	86.1	5.6
FHS incl original cohort	2313	10.5 (244)	57.6	54.0	7.9	27.9	92.0	81.2	10.9
KORA S3/F3 GWAS	1588	9.6 (153)	52.3	50.1	4.3	38.2	92.6	84.6	10.0
KORA S4/F4 GWAS	1737	5.3 (92)	53.4	51.2	3.4	33.4	90.8	86.1	7.1
KORA S3/F3 denovo	1235	3.3 (40)	40.5	51.7	1.6	22.7	99.4	94.2	9.7
KORA S4/F4 denovo	1149	4.1 (47)	41.1	52.6	1.7	20.7	98.9	93.9	7.2
Rotterdam Study	2236	12.6 (283)	66.6	58.6	7.9	49.5	79.5	74.5	6.4
SHIP	3084	5.3 (165)	49.2	51.8	11.2	53.1	92.4	90.6	5.3

Of the 16 SNPs analyzed, 11 were associated with incident CKD (Table 2): SNPs in *UMOD, PRKAG2, ANXA9, DAB2, SHROOM3, DACH1, STC1, SLC34A1, ALMS1/NAT8, UBE2Q2* and *GCKR* showed p-values ranging from p = 4.1×10^−9^ in *UMOD* to p = 0.03 in *GCKR.* The odds ratios (OR) for incident CKD of the minor alleles at each of the 11 loci ranged from 0.76 per copy of the T allele (allele frequency 18%) at the *UMOD* locus to 1.19 per copy of the A allele (allele frequency 22%) at *PRKAG2*. After additional adjustment for baseline eGFR, 6 SNPs (at the *UMOD, PRKAG2, ANXA9, DAB2, DACH1* and *STC1* loci) remained significantly associated with incident CKD, with minimal attenuation of effect size (Table 2).

**Table 2:** Results for incident CKD and ESRD, CKDGen consortium.

SNP ID	Locus ^#^	Chromo-some	Effect allele	Effect allele frequency	OR Incident CKD	incident CKD p-value	OR incident CKD adjusted for baseline-eGFR	incident CKD baseline-eGFR adjusted p-value	OR ESRD	ESRD p-value
rs12917707	*UMOD;FLJ20581,GP2,PDILT*	16	T	0.18	0.76	**4.1E-09**	0.79	**6.40E-07**	0.92	**0.04**
rs7805747	*PRKAG2*	7	A	0.22	1.19	**0.0004**	1.12	**0.01**	1.05	0.12
rs267734	*ANXA9;FAM63A,PRUNE,BNIPL,LASS2,SETDB1*	1	C	0.21	0.87	**0.001**	0.89	**0.005**	1.02	0.63
rs11959928	***DAB2*** *;C9*	5	A	0.45	1.10	**0.002**	1.07	**0.04**	0.94	0.94
rs17319721	***SHROOM3*** *;FLJ25770*	4	A	0.43	1.09	**0.005**	1.05	0.10	1.01	0.42
rs626277	*DACH1*	13	C	0.4	0.91	**0.006**	0.91	**0.006**	0.99*	0.56*
rs10109414	*STC1*	8	T	0.41	1.09	**0.006**	1.07	**0.04**	1.04	0.22
rs6420094	***SLC34A1*** *;GRK6,RGS14,LMAN2,PRR7,F12,PFN3*	5	G	0.34	1.10	**0.008**	1.05	0.13	0.96	0.87
rs13538	***NAT8*** *;NAT8B,ALMS1*	2	G	0.22	0.90	**0.009**	0.95	0.10	1.00	0.52
rs1394125	***UBE2Q2*** *;FBXO22*	15	A	0.34	1.08	**0.03**	1.06	0.07	0.94	0.91
rs1260326	***GCKR*** *;IFT172,FNDC4*	2	T	0.42	0.94	**0.03**	0.96	0.13	0.93	**0.03**
rs12460876	***SLC7A9*** *;CCDC123,ECAT8*	19	C	0.39	0.95	0.06	0.99	0.43	1.01	0.57
rs653178	***ATXN2,*** * BRAP*	12	T	0.5	0.95	0.08	0.97	0.20	1.05	0.87
rs4744712	***PIP5K1B*** *;FAM122A*	9	A	0.39	1.03	0.16	1.01	0.44	1.11*	0.07*
rs881858	*VEGFA*	6	G	0.29	0.97	0.22	1.03	0.78	0.98	0.28
rs347685	*TFDP2, ATP1B3*	3	C	0.28	1.01	0.57	1.05	0.13	1.05	0.83

Significant p-values in **bold**.

#The gene closest to the SNP is listed first and printed in bold if the SNP is located within the gene. Other genes in the region are listed after “;”.

*OR and p-value from random effects model due to significant heterogeneity between studies (rs4744712: p = 0.04 for heterogeneity; rs626277: p = 0.02 for heterogeneity).

At each of the significant loci, the direction and the magnitude of the association was similar to those from the discovery analyses of eGFR and prevalent CKD [17]. For example, at the *UMOD* locus, each copy of the minor T allele at rs12917707 was associated with a 24% reduced risk for incident CKD, while in the CKDGen consortium the same allele was associated with higher eGFR [17]. Though the associations between incident CKD and SNPs in *SLC7A9, ATXN2, PIP5K1B* and *VEGFA* were not significant, the direction and magnitude of associations were consistent with our previous findings for the phenotypes eGFR and prevalent CKD [16], [17]. *TFDP2* was the only locus where we did not observe association with incident CKD. Of the 16 SNPs tested, 15 had the same direction of association with incident CKD as their original associations with prevalent CKD. The probability of observing this many SNPs with consistency in direction of associations is 0.0002. We did not observe evidence for heterogeneity between studies at any of the 16 loci (test for heterogeneity p>0.05 for all SNPs).

### Association of SNPs with ESRD

For the ESRD analysis, we included four case-control studies with a total of 3775 ESRD patients and 4577 controls of European descent without CKD (Table 3). Mean age ranged from 50.7 to 66.2 years in cases and from 47.7 to 62.1 years in controls. Although the direction and magnitude of association for 8 SNPs (at the *UMOD, GCKR*, *PIP5K1B*, *PRKAG2*, *STC1*, *VEGFA*, *SHROOM3*, and *ALMS1/NAT8* loci) were consistent with our previous findings for eGFR and prevalent CKD [16], [17], only two SNPs showed nominally significant associations with ESRD (Table 2): rs1260326 in *GCKR* (OR =  0.93; p-value = 0.03) and rs12917707 in *UMOD* (OR =  0.92; p-value = 0.04). The lack of association was not likely due to heterogeneity of ESRD cases as only two SNPs showed moderate heterogeneity in their associations with ESRD (Table 2): rs4744712 at the *PIP5K1B* locus (p = 0.04 for heterogeneity) and rs626277 at the *DACH1* locus (p = 0.02 for heterogeneity).

**Table 3:** Characteristics of the ESRD case-control studies (n = 3,775 cases, n = 4,577 controls).§

		n	Mean Age (yrs)	Women (%)	DM (%)	HTN (%)
**ESRD cases**	GENDIAN	453	64.8	45.9	100.0	11.0
	4D	1148	65.7	45.7	100.0	89.0
	ArMORR	1244	66.2	47.8	23.6	39.9
	CHOICE	518	59.0	42.5	45.8	13.3
	FHKS	331	58.3	37.8	34.7	95.0
	MMKD	81	50.7	37.0	0.0	96.3
**Controls**	GENDIAN	326	62.1	43.3	100.0	32.2
	KORA F3 denovo	1407	50.4	52.7	4.4	27.8
	KORA F4 denovo	1130	47.7	52.3	3.2	12.2
	SAPHIR	1714	51.3	36.6	3.3	56.0

**§:** The four case-control studies comprised the following comparisons: GENDIAN cases versus GENDIAN controls, 4D versus KORA F3 denovo, ArMORR and CHOICE versus KORA F4 denovo, FHKS and MMKD versus SAPHIR.

## Discussion

Among individuals of European Ancestry, most genetic loci associated with the quantitative trait eGFR are also associated with risk for initiation of CKD, with more than half of these associations independent of eGFR at the baseline examination. In contrast, only two SNPs were nominally associated with ESRD.

To date, the genetic loci showing significant and replicated associations with ESRD are limited [13]–[15], [19]–[26], and genetic studies for incident CKD or for renal function decline in established kidney disease are only recently emerging [27]–[29]. The loci we analyzed were identified in association with renal function cross-sectionally and with prevalent CKD by GWAS in the general population. Typical of many SNPs uncovered in GWAS, the majority of these SNPs reside in intronic regions with unknown functional consequences, although several are associated with *cis* expression levels in liver tissue or leukocytes (Table S3) [16], [17]. These newly identified loci are non-overlapping with those previously identified in individuals of European or Asian descent with advanced diabetic nephropathy [19]–[26], or in African Americans with non-diabetic ESRD [13]–[15].

For the ESRD analysis, we had adequate power to detect effects that were similar to those for prevalent CKD in the discovery GWAS, where odds ratios ranged from 0.8 to 1.19 [16], [17]. In the present study, where associations were observed, the odds ratios for ESRD tended to be smaller and ranged from 0.92 to 1.11. There are several potential explanations for this effect dilution. First, the mechanisms involved in the initiation of CKD, the progression of CKD, and the incidence of ESRD may differ [30]–[33]. Experimental animal data and gene expression profiling in human kidney biopsies suggest differential biological pathways contributing to kidney disease initiation and progression [34]–[36]. Second, the majority of patients with CKD die of cardiovascular disease before developing ESRD [37]–[39]. Thus, the genetic findings for kidney function in the general population may not apply to the highly selected group of dialysis populations. Finally, the process of progression from CKD to ESRD often involves repeated insults including episodes of acute kidney injury by diagnostic and operative procedures and therapies [40]–[43], cardiac function deterioration [44], variation in access to adequate health care [45], [46] and other non-genetic factors [47]. Jointly, these factors may further decrease the relative impact of the small effects of SNPs derived from GWAS of eGFR in the general population at the earliest stage of disease initiation.

The observed small effect sizes for ESRD in our study are in contrast to the large effect sizes observed in relatively small cohorts of individuals of African descent for variants in the *MYH9/APOL1* locus, where odds ratios for ESRD ranged from 7.3 for the G1–G2 haplotype at the *APOL1* locus to 2.38 for the E1 haplotype in the *MYH9* locus [13]–[15]. However, the strong effect at this locus is an exceptional case and may be a consequence of a pronounced positive selection against vulnerability for *Trypanosoma brucei rhodesiense* infection at the price of a higher susceptibility for non-diabetic ESRD in African Americans not observed in other ethnicities. The establishment of large cohorts is thus needed for performing GWAS of CKD initiation and progression as well as ESRD to overcome the challenge of identifying novel loci significantly associated with these phenotypes with small effect sizes.

The strength of our work lies in the large number of individuals studied. Further, we exclusively analyzed candidate SNPs identified by the unbiased method of GWAS [16], [17]. However, some limitations warrant mention. First, seven of the eight cohorts used for the incident CKD analysis were also part of the CKDGen discovery effort; thus the two samples are not entirely “independent”. However, the phenotype studied differs substantially: in Köttgen et al [17], we used prevalent eGFR data including those with CKD, while follow-up data in those without CKD at the baseline examination was used for the present incident CKD analysis. In the present work, we demonstrate robustness of our findings independent of baseline GFR. Second, we relied on only two serum creatinine measurements to define incident CKD, which may have introduced misclassification and biased our findings towards the null. Third, we did not account for pharmacological treatment with inhibitors of the renin-angiotensin-aldosterone system. Since these drugs may affect kidney function independently of kidney damage, their use may have diluted observable genetic effects [48]. Fourth, our study was not designed to detect fluctuations in eGFR. Furthermore, the etiology of ESRD in the cases we examined may vary between studies, though we observed a low degree of heterogeneity. Finally, our sample consisted of individuals of European ancestry; findings may not be generalizable to other ethnicities.

SNPs associated with eGFR in population-based studies are associated with incident CKD, whereas modest associations were observed with ESRD. Additional work is necessary to characterize the genetic underpinnings across the full range of kidney disease phenotypes, which could ultimately lead to novel diagnostic and therapeutic strategies.

## Materials and Methods

### Ethics statement

In all studies, all participants gave informed consent. All studies were approved by their appropriate Research Ethics Committees.

### Study design and phenotype definition

In population based cohorts, serum creatinine measurements were calibrated to the National Health and Nutrition Examination Study (NHANES) standards in all studies to account for between-laboratory variation across studies, as described previously [10], [16], [17]. Using calibrated serum creatinine, we calculated the estimated glomerular filtration rate (eGFR) with the 4-variable MDRD equation [49].

For incident CKD, we analyzed studies of incident CKD in eight population-based cohorts in the CKDGen consortium with follow-up available: ARIC, CHS, CoLaus, FHS, KORA S3/F3, KORA S4/F4, the Rotterdam Study and SHIP. Each study's design is shown in Text S1. Incident CKD cases were defined as those free of CKD at baseline (defined as eGFR≥60 ml/min/1.73m^2^) but with a follow-up eGFR<60 ml/min/1.73m^2^. Controls were those free of CKD at baseline and at follow-up.

For the ESRD analysis, we performed four case control studies of ESRD. Cases were ESRD patients from six cohorts of ESRD patients: CHOICE, ArMORR, GENDIAN, 4D, MMKD and FHKS. Controls were those free of CKD (defined as eGFR≥60 ml/min/1.73m^2^) in three population-based cohorts (KORA F3, KORA F4, SAPHIR) and one type 2 diabetes cohort (GENDIAN). Each study's design is shown in Text S1.

### Statistical methods

In each study, we performed age- and sex adjusted logistic regression of incident CKD, with and without additional adjusting for baseline eGFR, or ESRD status with each SNP. In multicenter studies further adjustment for study-center was performed to account for possible differences between recruiting centers. For family-based studies, we applied logistic regression via generalized estimating equations (GEE) to account for the familial relatedness. Study-specific results were then combined by meta-analysis using a fixed effects model, using METAL (http://www.sph.umich.edu/csg/abecasis/Metal/index.html) [50]. When significant heterogeneity between studies was observed (p for heterogeneity between studies <0.05) we used the random effects model [51]. Statistical significance was defined as a one-sided p-value <0.05 for each SNP without adjustment for multiple testing since all SNPs examined had strong prior probabilities of being associated with the outcomes and the same alleles were hypothesized to be associated with lower eGFR, incident CKD, and ESRD.

### Power estimation

We used the QUANTO software for power estimation, assuming an additive genetic model (http://hydra.usc.edu/GxE) [52]. For the ESRD analysis and for SNPs with minor allele frequency ranging from 0.2 to 0.4 we had 80–100% power to detect an OR ≥ 1.10, whereas power was borderline for an OR of 1.05 to 1.09. For example, for the SNP rs12917707 at *UMOD*, we had 100% power to detect an association with ESRD in the 3775 ESRD cases and 4577 controls assuming that the effect in ESRD would be the same or larger than the effect observed for prevalent CKD previously [16], [17].

### Genotyping methods and quality control

For the incident CKD analysis, we used the allele dosage information of each of the 16 SNPs from each study's genome wide data set imputed to HAPMAP CEU samples described previously [17], [18]. Imputation provides a common SNP panel across all studies to facilitate a meta-analysis across all contributing SNPs. Information on each study's genotyping and imputation platform and quality control procedures are shown in Table S1. Table S2 summarizes each SNPs imputation quality.

De novo genotyping of the 16 SNPs was performed in each of the ESRD case-control studies as described previously [17]. Briefly, genotyping was performed either on a MassARRAY system using Assay Design v.3.1.2 and the iPLEX™ chemistry (Sequenom, San Diego, USA) at the Helmholtz Zentrum in Munich, Germany (ArMORR, GENDIAN, 4D, MMKD, FHKS, KORA S3/F3-subset without GWAS data, KORA S4/F4-subset without GWAS data, SAPHIR); by using 5′ nuclease allelic discrimination assays on 7900HT Fast Real-Time Taqman PCR genotyping systems (Applied Biosystems, Foster City, CA, USA) at the Innsbruck Medical University (ArMORR, GENDIAN, 4D, MMKD, FHKS, KORA F3-subset without GWAS data, KORA F4-subset without GWAS data, SAPHIR); or as part of a larger panel of 768 SNPs genotyped on the Illumina Bead Station (CHOICE). The SNPs rs347685, rs11959928, rs4744712 and rs12460876 were not available for de novo genotyping on the Sequenom platform, thus the proxy SNPs rs6773343, rs11951093, rs1556751 and rs8101881, with pairwise r^2^ of 1.0, 0.87, 0.87 and 1.0 respectively [53], were included in the MassARRAY multiplex PCR.

For the obtained duplicate genotypes (9–22% of the subjects in GENDIAN, 4D, MMKD, FHKS, KORA F3-subset without GWAS data, KORA F4-subset without GWAS data, and SAPHIR; no duplicate genotyping possible due to limited DNA-availability in CHOICE and ArMORR) concordance was 96–100% (median: 100%). SNPs with a per-study call rate <90% or with a per-study HWE p value <0.0001 were excluded from further analysis (rs6773343 and rs653178 in GENDIAN cases; rs13538, rs267734, rs10109414, rs1394125 in ArMORR, rs6773343, rs10109414, rs1556751, rs653178, rs8101881 in CHOICE). In addition, individual samples with <80% successfully genotyped SNPs were excluded from further analysis. After these exclusions, call rates ranged from 91–100% (mean: 98%) across all studies and all SNPs.

## Supporting Information

Table S1 Genotyping and Imputation Platforms Used by Studies in the incident CKD analysis.
(DOC)

Table S2 Imputation quality scores of SNPs across incident CKD cohorts.
(DOC)

Table S3 Location and function of analyzed SNPs.
(DOC)

Text S1 Study-specific details.
(DOC)
