# Supplementary material for: Association of eGFR-Related Loci Identified by GWAS with Incident CKD and ESRD
Source: PLoS Genet. 2011 Sep 29;7(9):e1002292. doi: 10.1371/journal.pgen.1002292 (PMC3183079; doi:10.1371/journal.pgen.1002292)
Supplement: Table S1 — Genotyping and Imputation Platforms Used by Studies in the incident CKD analysis. (DOC) [file pgen.1002292.s001.doc]

| **Table S1: Genotyping and Imputation Platforms Used by Studies in the incident CKD analysis** | | | | |  |  |  |  |
| --- | --- | --- | --- | --- | --- | --- | --- | --- |
|  | **Array type** | **Genotype calling** | **QC filters for genotyped SNPs used for imputation** | **No of SNPs used for imputation** | **Imputation** | **Imputation Backbone (NCBI build)** | **Filtering of imputed genotypes** | **Data management and statistical analysis** |
| **ARIC** | Affymetrix 6.0 | Birdseed | call rate <95%, MAF<1%, pHWE<10E-5 | 704588 | MACH version 1.0.16 | phased CEU haplotypes, HapMap release 21 (build 35) | none | Linear and logistic regression using ProbABEL, PLINK, R |
| **CHS** | Illumina 370CNV | Illumina BeadStudio | call rate <97%, heterozygotes=0, pHWE<10E-5, SNP not in HapMap | 306655 | BimBam | HapMap CEU release 21A (build 36) | dosage variance < 0.01 | Linear and logistic regression using R |
| **CoLaus** | Affymetrix 500K | BRLMM (Affymetrix) | call rate <90%, pHWE<1E-7, MAF<1% | 390631 | IMPUTE version 0.2.0 | HapMap release 21 (build 35) | none | Matlab |
| **FHS** | Affymetrix 500K  Affymetrix 50K supplemental | Affymetrix | pHWE<1e-6,call rate<97%,mishap p<1e-9,MAF<0.01,Mendelian errors>100, SNPs not in Hapmap or strandedness issues merging with Hapmap | 378163 | MACH version 1.0.15 | phased CEU haplotypes, HapMap release 22 (build 36) | none | R |
| **KORA S3/F3** | Affymetrix 500K | BRLMM | per-chip call rate <93%, MAF <5%, discrepancy for one of the 50 SNPs common on both chips, gender checks | 380407 | MACH | HapMap CEU sample, b35 | none | MACH2QTL, PROBABEL, R, Visual basic |
| **KORA S4/F4** | Affymetrix 6.0 | BRLMM | per-chip call rate <93%, per-SNP call rate <93%, MAF<1%, gender checks | 629893 | MACH | HapMap CEU samples, b36 | none | PROBABEL, R, Visual basic |
| **RS-I** | Illumina 550K | Illumina Beadstudio | call rate <90%, MAF<1%, pHWE<10E-5 | 530683 | MACH version 1.0.15 | phased CEU haplotypes, HapMap release 22 (build 36) | none | Linear and logistic regression using ProbABEL, R |
| **SHIP** | Affymetrix Genome-Wide SNP 6.0 | Birdseed V2 | per-chip call rate < 86%, per-SNP call rate < 92% | 869224 | IMPUTE v0.5.0 | phased CEU haplotypes, HapMap release 22 (build 36) | none | SNPTEST v1.1.5 |
