# Supplementary material for: Association of eGFR-Related Loci Identified by GWAS with Incident CKD and ESRD
Source: PLoS Genet. 2011 Sep 29;7(9):e1002292. doi: 10.1371/journal.pgen.1002292 (PMC3183079; doi:10.1371/journal.pgen.1002292)
Supplement: Table S2 — Imputation quality scores of SNPs across incident CKD cohorts. (DOC) [file pgen.1002292.s002.doc]

| **Table S2: Imputation quality scores of SNPs across incident CKD cohorts** | | | |  |  |  |  |  |  |  |
| --- | --- | --- | --- | --- | --- | --- | --- | --- | --- | --- |
| **SNP ID** | **Locus^#^** | **ARIC** | **CHS** | **CoLaus** | **FHS** | **KORA S3/F3** | **KORA S4/F4** | **Rotterdam Study** | **SHIP** | **median imputation quality** |
| rs267734 | *ANXA9;FAM63A,PRUNE,BNIPL,LASS2,SETDB1* | 1.00 | 0.95 | 0.99 | 0.98 | 1.00 | 1.00 | 0.99 | 1.00 | **1.00** |
| rs1260326 | ***GCKR****;IFT172,FNDC4* | 0.98 | 1.01 | 0.98 | 0.99 | 0.96 | 0.96 | 0.96 | 0.99 | **0.98** |
| rs13538 | ***NAT8****;NAT8B,ALMS1* | 1.00 | 0.45 | 0.99 | 0.99 | 0.95 | 0.98 | 0.99 | 0.99 | **0.99** |
| rs347685 | *TFDP2, ATP1B3* | 1.00 | 1.02 | 1.00 | 1.03 | 1.00 | 1.00 | 1.00 | 1.00 | **1.00** |
| rs17319721 | ***SHROOM3****;FLJ25770* | 1.00 | 0.74 | 1.00 | 1.01 | 0.96 | 1.00 | 0.99 | 1.00 | **1.00** |
| rs11959928 | ***DAB2****;C9* | 0.98 | 0.87 | 0.95 | 0.99 | 0.89 | 0.93 | 0.98 | 0.99 | **0.97** |
| rs6420094 | ***SLC34A1****;GRK6,RGS14,LMAN2,PRR7,F12,PFN3* | 0.61 | 0.99 | 0.50 | 0.48 | 0.51 | 0.62 | 1.00 | 0.70 | **0.62** |
| rs881858 | *VEGFA* | 0.95 | 0.83 | 0.67 | 0.69 | 0.73 | 0.93 | 0.88 | 0.95 | **0.86** |
| rs7805747 | ***PRKAG2*** | 0.57 | 0.43 | 0.53 | 0.50 | 0.54 | 0.52 | 0.92 | 0.51 | **0.52** |
| rs10109414 | *STC1* | 0.99 | 0.85 | 0.99 | 0.99 | 1.00 | 0.98 | 0.99 | 1.00 | **0.99** |
| rs4744712 | ***PIP5K1B****;FAM122A* | 1.00 | 0.96 | 1.00 | 1.00 | 1.00 | 0.98 | 1.00 | 0.99 | **1.00** |
| rs653178 | ***ATXN2,*** *BRAP* | 0.99 | 1.05 | 0.83 | 0.99 | 0.79 | 1.00 | 1.00 | 1.00 | **1.00** |
| rs626277 | ***DACH1*** | 0.99 | 0.98 | 0.79 | 0.78 | 0.58 | 1.00 | 1.00 | 1.00 | **0.99** |
| rs1394125 | ***UBE2Q2****;FBXO22* | 0.68 | 1.01 | 0.66 | 0.71 | 0.54 | 0.67 | 0.94 | 0.79 | **0.70** |
| rs12917707 | *UMOD;FLJ20581,GP2,PDILT* | 0.94 | 0.96 | 0.92 | 0.96 | 0.86 | 0.87 | 0.98 | 0.99 | **0.95** |
| rs12460876 | ***SLC7A9****;CCDC123,ECAT8* | 0.99 | 0.87 | 0.94 | 0.98 | 0.93 | 0.94 | 1.00 | 0.98 | **0.96** |

# The gene closest to the SNP is listed first and printed in bold if the SNP is located within the gene. Other genes in the region are listed after ";".
