# Supplementary material for: Association of eGFR-Related Loci Identified by GWAS with Incident CKD and ESRD
Source: PLoS Genet. 2011 Sep 29;7(9):e1002292. doi: 10.1371/journal.pgen.1002292 (PMC3183079; doi:10.1371/journal.pgen.1002292)
Supplement: Table S3 — Location and function of analyzed SNPs. (DOC) [file pgen.1002292.s003.doc]

| **Table S3:** Location and function of analyzed SNPs. | | | |  |
| --- | --- | --- | --- | --- |
| **SNP ID** | **Chromo-some** | **Genes Within 60 kb** | **SNP function** | **Gene implicated**  **by eSNP analysis [17,54-56]*** |
| rs267734 | 1 | *ANXA9;FAM63A,PRUNE,BNIPL,LASS2,SETDB1* | Intergenic | -- |
| rs1260326 | 2 | ***GCKR****;IFT172,FNDC4* | non-synonymous coding | *IFT172;*  *GCKR* (r^2^=0.03) |
| rs13538 | 2 | ***NAT8****;NAT8B,ALMS1* | non-synonymous coding | *ALMS1 (*r^2^=1.0)  *NAT8* (r^2^=0.42); *TPRKB* (r^2^=0.39) |
| rs347685 | 3 | *TFDP2, ATP1B3* | intergenic | *ATP1B3* (r^2^=0.67) |
| rs17319721 | 4 | ***SHROOM3****;FLJ25770* | intronic | *SHROOM3* (r^2^=0.06) |
| rs11959928 | 5 | ***DAB2****;C9* | intronic | *DAB2 (*r^2^*=0.93)* |
| rs6420094 | 5 | ***SLC34A1****;GRK6,RGS14,LMAN2,PRR7,F12,PFN3* | intronic | *--* |
| rs881858 | 6 | *VEGFA* | intergenic | *--* |
| rs7805747 | 7 | ***PRKAG2*** | intronic | *--* |
| rs10109414 | 8 | *STC1* | intergenic | *C2orf29* (r^2^=0.29) |
| rs4744712 | 9 | ***PIP5K1B****;FAM122A* | intronic | *FAM122A* (r^2^=0.19) |
| rs653178 | 12 | ***ATXN2,*** *BRAP* | intronic | *--* |
| rs626277 | 13 | ***DACH1*** | intronic | *--* |
| rs1394125 | 15 | ***UBE2Q2****;FBXO22* | intronic | *UBE2Q2* (r^2^=0.01), *FBXO22* (r^2^=0.01) |
| rs12917707 | 16 | *UMOD;FLJ20581,GP2,PDILT* | upstream | *--* |
| rs12460876 | 19 | ***SLC7A9****;CCDC123,ECAT8* | intronic | *SLC7A9* (r^2^=0.58) |

The gene closest to the SNP is listed first and printed in bold if the SNP is located within the gene. Other genes in the region are listed after ";".

* Where the eSNP is not identical to the index SNP, the r^2^ is given for the correlation of eSNP and index SNP.
