# Supplementary material for: Association of eGFR-Related Loci Identified by GWAS with Incident CKD and ESRD
Source: PLoS Genet. 2011 Sep 29;7(9):e1002292. doi: 10.1371/journal.pgen.1002292 (PMC3183079; doi:10.1371/journal.pgen.1002292)
Supplement: Text S1 — Study-specific details. (DOC) [file pgen.1002292.s004.doc]

**SUPPORTING INFORMATION**

**Association of eGFR-related loci identified by GWAS
with incident CKD and ESRD**

**Supplementary Methods**

1. Study specific details

*1.a) incident CKD analysis*

**Atherosclerosis Risk in Communities (ARIC)**: The ARIC Study is a prospective, population-based cohort of 15,792 adults, aged 45 to 64 years at the baseline visit (1987-1989). Participants were recruited from four US communities and underwent four standardized examinations approximately every 3 years [57]. Institutional review boards of participating institutions approved the study protocols. Serum creatinine was measured using a modified kinetic Jaffe reaction. Serum creatinine from visits 1, 2, and 4 were used to estimate eGFR and define CKD. Individuals with eGFR < 60 ml/min per 1.73 m^2^ at visit 1 were considered to have prevalent CKD and were excluded from the present study. Among individuals of European descent free of CKD at baseline, incident CKD was defined having a eGFR < 60 ml/min per 1.73 m^2^ at either visit 2 or 4 of the study.

**Cardiovascular Health Study (CHS)**: The Cardiovascular Health Study (CHS) is a population based longitudinal study of risk factors for cardiovascular disease and stroke in adults aged 65 years or older, recruited at four field centers (Forsyth County, NC; Sacramento County, CA; Washington County, MD; Pittsburgh, PA) [58]. A total of 5201 individuals of predominantly European ancestry were recruited in 1989-1990 from random samples of Medicare eligibility lists. Follow-up examinations including determination of serum creatinine were performed in 1989-1990 (n=5165), 1992-1993 (n=4049) and 1996-1997 (n=2954). The serum creatinine at baseline and at the last two follow-ups were used to define the longitudinal renal phenotypes. After exclusion of persons with non-European ancestry, coronary heart disease, congestive heart failure, peripheral vascular disease, valvular heart disease, stroke or transient ischemic attack or lack of available DNA at baseline, a total of n=2389 subjects with complete follow-up and genome wide data was available for the current analysis.

Serum creatinine was measured by a colorimetric method (Ektachem 700, Eastman Kodak). Covariates were obtained at baseline.

**Cohorte Lausannoise (CoLauS)**

Cohorte Lausannoise (CoLaus) is a population-based study aimed at assessing the prevalence and molecular determinants of cardiovascular risk factors in the Caucasian population of Lausanne, Switzerland [59]. Participants in the study were randomly selected from the population register of Lausanne in 2003 (n=56,694, aged 35-75 years). All individuals were of Caucasian origin, defined as having both parents and grandparents born in a defined list of European countries.

The 6184 participants available at baseline (2003-2006) are being followed-up at 6 year (2009-2011). A total of 75 cases and 1767 controls for the CKD analysis had complete baseline, follow-up and genotyping data, with mean follow-up of 5.6 years.

Covariates were obtained at the baseline examination. Serum creatinine was measured by the Jaffe kinetic compensated method (2.9% – 0.7% maximum inter and intra-batch CVs) on fasting samples.

**Framingham Heart Study (FHS)**

The Framingham Heart Study began in 1948 with enrollment of the Original Cohort [60]. Enrollment of the second generation of participants, the Offspring Cohort, began in 1971 (5124 participants); the methodology and design has previously been described [61,62]. In 2002, the Third Generation cohort was enrolled (n=4095) [63]. Participants for the current study include individuals of European descent from the original cohort who attended cohort exam 15 (1977 to 1979) and exam 24 (1995 to 1998) [n=548], as well as participants from the offspring cohort who attended the sixth (1995 – 1998) and the eighth exams (2005-2008) [n=2234]. Incident CKD was defined as cases of CKD (eGFR <60 ml/min/1.73m^2^) present at exam cycle 24 (original cohort) and exam cycle 8 (offspring cohort) in individuals free of CKD at the earlier respective exam (i.e. exam 15 for the original cohort and exam 6 for the offspring cohort). In total 2523 participants had complete baseline and follow-up information, and were successfully genotyped for GWA analysis. Covariates were obtained at the index examination. Serum creatinine in FHS was measured using a modified Jaffé reaction.

**KORA cohorts**

The KORA surveys for genetic research have been described in detail previously and were initiated as part of the MONICA (Monitoring of Trends of Cardiovascular Diseases) multi-center study [3,64]. All participants of the KORA cohorts had a German passport and are of European origin. The third KORA survey (KORA S3) is a population-based sample recruited 1994/1995 from the general population of the South-German city of Augsburg and surrounding counties. A total of 1641 individuals from this survey with complete baseline and 10-year follow-up (KORA F3) information were successfully genotyped for GWA analysis. A total of 1498 subjects from the third KORA survey who had not been genotyped genome wide were included for de novo genotyping of the 16 SNPs. Of these, 1274 subjects had serum creatinine measured at both examinations (S3 and F3), and were thus available for the incident CKD analyses. The fourth KORA survey (KORA S4) is a sample recruited 1999-2001 independent from KORA S3 using the same platform with the same standard operating procedures and based on the same population. A subset totaling 1807 subjects with complete baseline and 7-year follow-up (KORA F4) was successfully genotyped for GWA analysis. A total of 1202 subjects from the fourth KORA survey without genome wide genotype data were included for de novo genotyping of the 16 SNPs. Of these, 1191 individuals had serum creatinine measured at both examinations (S4 and F4).

For the incident CKD analyses, only those with eGFR ≥60 ml/min/1.73m^2^ at the baseline examinations were considered; for the ESRD analyses, only those with eGFR ≥60 ml/min/1.73m^2^ at the follow-up examinations were considered. Covariates were obtained at the index examination. Serum creatinine in KORA F3 and F4 was measured using a modified kinetic Jaffé reaction. Serum creatinine in KORA S3 and S4 were measured using an enzymatic assay.

**The Rotterdam Study**

The Rotterdam Study is a prospective, population-based cohort study on determinants of several chronic diseases [65-67]. Briefly, 10275 inhabitants of Ommoord, a district of Rotterdam in the Netherlands, who were 55 years or over, were invited to participate in this study and 7983 agreed to participate (78%). The baseline examination (1989-1993) was composed of a home interview and a visit to the research center for blood sampling and examination. A total of 5974 individuals were successfully genotyped for GWA analysis. Creatinine levels were measured for 4390 individuals at baseline and 4006 subjects at the third periodical visit (1997-1999). Serum creatinine was measured using a modified kinetic Jaffe reaction at baseline [68] and an enzymatic assay in the third periodical visit. Covariates were obtained at the baseline examination.

**The Study of Health in Pomerania (SHIP)**

The Study of Health in Pomerania (SHIP) is a longitudinal population-based cohort study conducted in West Pomerania, the north-east area of Germany [69,70]. For the baseline examinations, a sample of 6.267 eligible subjects aged 20 to 79 years was drawn from population registries. Only individuals with German citizenship and main residency in the study area were included. SHIP finally comprised 4,308 participants corresponding to a final response rate of 68.7%.

Baseline examinations were conducted between 1997 and 2001. Between 2002 and 2006 all participants were re-invited for an examination follow-up, in which 3,300 subjects (83.5% of eligible persons) took part. The final population for the present study consisted of 3,084 subjects with complete GWAS and renal function data.

Covariates were obtained at the baseline examination. Serum creatinine levels were determined according to the Jaffé method.

*1.b) ESRD analysis*

**ArMORR**

ArMORR (Accelerated Mortality on Renal Replacement) is a prospective cohort study. This study included 10,044 incident hemodialysis patients, who started chronic hemodialysis in 2004 or 2005, at any of 1056 dialysis centers operated by Fresenius Medical Care, North America (Waltham, MA) [71]. All clinical data and blood samples were collected at the point of care. After routine tests were processed, residual blood samples of 2490 random subjects from the study were used for DNA extraction, establishing the ArMORR DNA Repository [72]. These subjects with available DNA were initially included in the current study.

After exclusion of non-Caucasian individuals and of DNA with less than 80% successfully genotyped SNPs, 1244 subjects were included for the current analyses.

**CHOICE**

The CHOICE (Choices for Healthy Outcomes In Caring for ESRD) study is a prospective cohort study of 1,041 adult dialysis patients enrolled between 1995 and 1998 from 81 U.S. dialysis clinics associated with Dialysis Clinic, Inc within a median of 45 days after initiation of dialysis (98% within 4.0 months), described previously [73]. Demographic data was collected through a self-administered questionnaire. Comorbid conditions such as hypertension (blood pressure >140/90 mmHg) and diabetes mellitus were abstracted from charts. DNA was available in 824 participants, and only individuals with self-reported Caucasian ethnicity were included in the present study. The study was approved by the Institutional Review Board at Johns Hopkins University School of Medicine, and participants provided written informed consent.

**Mild to Moderate Kidney Disease (MMKD) Study**

We examined at baseline 227 Caucasian patients between the ages of 18 and 65 years with non-diabetic CKD and various degrees of renal impairment. These individuals were recruited from 8 nephrology departments in Germany, Austria, and South Tyrol (Italy) as described earlier [74,75]. The study was approved by the Institutional Ethic Committee and all participants gave their informed consent before inclusion in the study.

The patients had stable renal function for at least three months before entry into the study. The primary cause of CKD was glomerulonephritis in 97 patients (biopsy-confirmed in 90), adult polycystic kidney disease in 37 patients, interstitial nephritis in 24 patients, other types of kidney disease in 43 patients, and unknown in 26 patients. Exclusion criteria were serum creatinine >6 mg/dL (531 μmol/L), diabetes mellitus of any type, malignancy, liver, thyroid or infectious diseases, nephrotic syndrome (defined as daily proteinuria >3.5 g/1.73 m^2^), organ transplantation, immunosuppressive treatment, allergy to ionic contrast media, treatment with fish oil or erythropoietin, and pregnancy. To avoid inter-observer differences, all patients were recruited by a single investigator who visited all participating centers.

Of the baseline cohort, a total of 177 patients (78%) were followed prospectively until 2006. For the present analysis only those patients with progression of kidney disease to ESRD (n=81), defined as the need for renal replacement therapy, were considered.

Laboratory procedures: Serum and EDTA plasma were taken after a 12h overnight fast. After low-speed centrifugation, samples were frozen and kept at -80°C prior to analysis. Antihypertensive medication (if present) was withheld on the day of the study in order to minimize interference with measurements of GFR. Patients were carefully instructed about the collection of a 24-hour urine sample for the determination of proteinuria. Measurement of serum creatinine and albumin as well as GFR by iohexol were performed each centrally in a single laboratory, to avoid interlaboratory differences in measurements. At this time the laboratory staff involved in the study was unaware of the patient's renal function. Serum creatinine (mg/dl) was measured using a Jaffe reaction standardized according to IDMS (Autoanalyzer Modular P from Roche Diagnostics GmbH, Mannheim, Germany). Cystatin C was measured by a nephelometric test from Dade Behring using a BN Prospec Nephelometer. GFR was determined by iohexol clearance: depending on the serum creatinine level, two to three blood samples were collected for the determination of GFR by the iohexol method [76] obtained after infusion of iohexol during the same visit in the outpatient department.

**Family Heart and Kidney Study (FHKS)**

The “Family Heart and Kidney Study” (FHKS) is an ongoing prospective multicenter cohort study that started in 2004 with the recruitment of the first patients. Its aim is to investigate the genetic variability of selected candidate genes influencing the HDL cholesterol and triglyceride metabolism as well as their influence on atherosclerotic complications in the high-risk group of hemodialysis patients.

Until now 331 patients of European descent have been recruited from eight renal units in Austria (Innsbruck, Feldkirch, Wilhelminenspital Wien, Wien-Hietzing, St. Pölten, Pörtschach, Zams) and Germany (Mettmann) where they attend dialysis treatment on average three times per week. These patients are prospectively followed. Patients aged from 25 to 75 years being on dialysis treatment since at least four weeks but no longer than three years are being included in the study independent of the underlying kidney disease. Exclusion criteria are former organ transplantations and malignancy.

Blood samples are taken after an overnight fast following the long dialysis time interval without having a serious disease during the last four weeks. We implemented a questionnaire survey to collect data to prevalent cardiovascular diseases and their risk factors, family history of cardiovascular disease as well as quality of life of the dialysis patients. The study was approved by the institutional ethics committees, and subjects gave written informed consent.

**GENDIAN**

GENDIAN (GENetics of DIAbetic Nephropathy) is a case control study of diabetic nephropathy in Caucasian subjects [77,78]. Cases are prevalent patients on maintenance hemodialysis due to diabetic nephropathy (n=477), recruited from 30 dialysis centers in Southern Germany between August 1999 and January 2000, and n=99 patients with biopsy proven diabetic nephropathy. A total of n=639 controls not on renal replacement therapy were recruited from 2000-2001 (n=482) and from 2004-2006 (n=67) in a large diabetes clinic in Southern Germany (Diabetes Zentrum Mergentheim), and in 2007 (n=90) in outpatient clinics in the greater Regensburg region. Follow-up examinations were performed in 2007 in all controls except those recruited in 2007. For the current analysis, cases were defined as those with ESRD due to diabetic nephropathy, controls as those with eGFR≥60ml/min/1.73m^2^ at the examination in 2007. After exclusion of n=24 cases and n=24 controls with missing DNA or < 80% successfully genotyped SNPs there remained n=453 cases and n=326 controls that entered the analyses.

Serum creatinine was measured using the CreaPlus enzymatic assay.

**4D**

The German Diabetes and Dialysis Study (4D study) methodology has previously been reported in detail [79]. Briefly, the 4D study was a prospective randomized controlled trial (RCT), including 1255 patients with type 2 diabetes mellitus of European descent, aged 18–80 years, and on haemodialysis for <2 years. Between March 1998 and October 2002, patients were recruited in 178 dialysis centres in Germany. After a period of 4 weeks, patients were randomly assigned to double-blinded treatment with either 20 mg of atorvastatin (n = 619) or placebo (n = 636) once daily. The primary study endpoint was a composite of death from cardiac causes, myocardial infarction (MI) and stroke, whichever occurred first.

For the current analysis, cases were defined as those with ESRD due to diabetic nephropathy. DNA for SNP analysis was available from 1148 patients.

**SAPHIR-Study**

The "Salzburg Atherosclerosis Prevention Program in subjects at High Individual Risk" (SAPHIR) is an observational study conducted in the years 1999-2002 involving healthy unrelated subjects of European descent: 641 females from 39 to 67 years of age and 1092 males from 39 to 66 years of age. Study participants were recruited by health screening programs in large companies in and around the city of Salzburg as described recently [80]. All individuals were of West-Eurasian origin. Subjects with established coronary artery, cerebrovascular or peripheral arterial disease, congestive heart failure, valvular heart disease, chronic alcohol (more than three drinks a day) or drug abuse, severe obesity (BMI>40kg/m²) and pregnant women were excluded. Informed consent was obtained from each participant. At baseline all study participants were subjected to a comprehensive screening examination. A detailed personal and family history was assessed via standardized questionnaires. A physical examination included measurement of anthropometric parameters such as weight, height, waist circumference and percentage body fat. Blood samples were collected after an overnight fasting period.

Among the total of 1733 phenotyped participants, a total of 1714 had eGFR higher or equal than 60 ml/min/1.73 m^2^ (defined as having no CKD) and were used as control group for diseased populations such as the Family Heart and Kidney Study (FHKS) and the Mild to Moderate Kidney Disease (MMKD) Study.

Serum and urinary creatinine (mg/dl) was measured using a modified kinetic Jaffe reaction (CREA®, Roche Diagnostics GmbH, Mannheim, Germany); eGFR was calculated using the Modification of Diet in Renal Disease (MDRD) Study equation [49].

Covariates were obtained at the index examination. Diabetes was defined as a fasting plasma glucose of at least 126 mg/dl (7.0 mmol/L) or treatment with a hypoglycemic agent. Hypertension was defined as systolic blood pressure of at least 140 mm Hg, diastolic blood pressure of at least 90 mm Hg, or pharmacologic treatment for hypertension.

**KORA F3 and KORA F4**

For the ESRD analysis, we performed de novo genotyping of the 16 eGFR-related SNPs in KORA F3 and KORA F4 samples without genome wide genotype data sets. Please see “KORA cohorts” in the incident CKD section above for further details on the KORA cohorts.

**References Supporting Information**

**References**

1. Levey AS, Atkins R, Coresh J, Cohen EP, Collins AJ, et al. (2007) Chronic kidney disease as a global public health problem: approaches and initiatives - a position statement from Kidney Disease Improving Global Outcomes. Kidney Int 72:247-59.

2. Matsushita K, van der Velde M, Astor BC, Woodward M, Levey AS, et al. (2010) Association of estimated glomerular filtration rate and albuminuria with all-cause and cardiovascular mortality in general population cohorts: a collaborative meta-analysis. Lancet 375:2073-81.

3. Baumeister SE, Böger CA, Krämer BK, Doring A, Eheberg D, et al. (2010) Effect of chronic kidney disease and comorbid conditions on health care costs: A 10-year observational study in a general population. Am J Nephrol 31:222-9.

4. Meguid El Nahas A, Bello AK (2005) Chronic kidney disease: the global challenge. Lancet 365:331-40.

5. Adler AI, Stevens RJ, Manley SE, Bilous RW, Cull CA, et al. (2003) Development and progression of nephropathy in type 2 diabetes: the United Kingdom Prospective Diabetes Study (UKPDS 64). Kidney Int 63:225-32.

6. Hsu CY, McCulloch CE, Darbinian J, Go AS, Iribarren C (2005) Elevated blood pressure and risk of end-stage renal disease in subjects without baseline kidney disease. Arch Intern Med 165:923-8.

7. Kastarinen M, Juutilainen A, Kastarinen H, Salomaa V, Karhapaa P, et al. Risk factors for end-stage renal disease in a community-based population: 26-year follow-up of 25,821 men and women in eastern Finland. J Intern Med 267:612-20.

8. Ritz E, Stefanski A (1996) Diabetic nephropathy in type II diabetes. Am J Kidney Dis 27:167-94.

9. Coresh J, Selvin E, Stevens LA, Manzi J, Kusek JW, et al. (2007) Prevalence of chronic kidney disease in the United States. JAMA 298:2038-47.

10. Fox CS, Larson MG, Leip EP, Culleton B, Wilson PW, et al. (2004) Predictors of new-onset kidney disease in a community-based population. JAMA 291:844-50.

11. Fox CS, Muntner P (2008) Trends in diabetes, high cholesterol, and hypertension in chronic kidney disease among U.S. adults: 1988-1994 to 1999-2004. Diabetes Care 31:1337-42.

12. Satko SG, Sedor JR, Iyengar SK, Freedman BI (2007) Familial clustering of chronic kidney disease. Semin Dial 20:229-36.

13. Genovese G, Friedman DJ, Ross MD, Lecordier L, Uzureau P, et al. (2010) Association of trypanolytic ApoL1 variants with kidney disease in African Americans. Science 329:841-5.

14. Kao WH, Klag MJ, Meoni LA, Reich D, Berthier-Schaad Y, et al. (2008) MYH9 is associated with nondiabetic end-stage renal disease in African Americans. Nat Genet 40:1185-92.

15. Kopp JB, Smith MW, Nelson GW, Johnson RC, Freedman BI, et al. (2008) MYH9 is a major-effect risk gene for focal segmental glomerulosclerosis. Nat Genet 40:1175-84.

16. Köttgen A, Glazer NL, Dehghan A, Hwang SJ, Katz R, et al. (2009) Multiple loci associated with indices of renal function and chronic kidney disease. Nat Genet 41:712-7.

17. Köttgen A, Pattaro C, Böger CA, Fuchsberger C, Olden M, et al. (2010) New loci associated with kidney function and chronic kidney disease. Nat Genet 42:376-84.

18. Chambers JC, Zhang W, Lord GM, van der Harst P, Lawlor DA, et al. (2010) Genetic loci influencing kidney function and chronic kidney disease. Nat Genet 42:373-5.

19. Ma RC, Tam CH, Wang Y, Luk AO, Hu C, et al. (2010) Genetic variants of the protein kinase C-beta 1 gene and development of end-stage renal disease in patients with type 2 diabetes. JAMA 304:881-9.

20. Shimazaki A, Kawamura Y, Kanazawa A, Sekine A, Saito S, et al. (2005) Genetic variations in the gene encoding ELMO1 are associated with susceptibility to diabetic nephropathy. Diabetes 54:1171-8.

21. Pezzolesi MG, Katavetin P, Kure M, Poznik GD, Skupien J, et al. (2009) Confirmation of genetic associations at ELMO1 in the GoKinD collection supports its role as a susceptibility gene in diabetic nephropathy. Diabetes 58:2698-702.

22. Pezzolesi MG, Poznik GD, Mychaleckyj JC, Paterson AD, Barati MT, et al. (2009) Genome-wide association scan for diabetic nephropathy susceptibility genes in type 1 diabetes. Diabetes 58:1403-10.

23. Alkhalaf A, Bakker SJ, Bilo HJ, Gans RO, Navis GJ, et al. A polymorphism in the gene encoding carnosinase (CNDP1) as a predictor of mortality and progression from nephropathy to end-stage renal disease in type 1 diabetes mellitus. Diabetologia 53:2562-8.

24. Freedman BI, Bostrom M, Daeihagh P, Bowden DW (2007) Genetic factors in diabetic nephropathy. Clin J Am Soc Nephrol 2:1306-16.

25. He B, Osterholm AM, Hoverfalt A, Forsblom C, Hjorleifsdottir EE, et al. (2009) Association of genetic variants at 3q22 with nephropathy in patients with type 1 diabetes mellitus. Am J Hum Genet 84:5-13.

26. Zhang D, Efendic S, Brismar K, Gu HF Effects of MCF2L2, ADIPOQ and SOX2 genetic polymorphisms on the development of nephropathy in type 1 Diabetes Mellitus. BMC Med Genet 11:116.

27. Köttgen A, Hwang SJ, Rampersaud E, Coresh J, North KE, et al. (2008) TCF7L2 variants associate with CKD progression and renal function in population-based cohorts. J Am Soc Nephrol 19:1989-99.

28. Liu M, Shi S, Senthilnathan S, Yu J, Wu E, et al. (2010) Genetic variation of DKK3 may modify renal disease severity in ADPKD. J Am Soc Nephrol 21:1510-20.

29. Wheeler HE, Metter EJ, Tanaka T, Absher D, Higgins J, et al. (2009) Sequential use of transcriptional profiling, expression quantitative trait mapping, and gene association implicates MMP20 in human kidney aging. PLoS Genet 5: e1000685. doi:10.1371/journal.pgen.1000685

30. Brancati FL, Whelton PK, Randall BL, Neaton JD, Stamler J, et al. (1997) Risk of end-stage renal disease in diabetes mellitus: a prospective cohort study of men screened for MRFIT. Multiple Risk Factor Intervention Trial. JAMA 278:2069-74.

31. (2002) K/DOQI clinical practice guidelines for chronic kidney disease: evaluation, classification, and stratification. Am J Kidney Dis 39:S1-266.

32. Bash LD, Astor BC, Coresh J (2010) Risk of incident ESRD: a comprehensive look at cardiovascular risk factors and 17 years of follow-up in the Atherosclerosis Risk in Communities (ARIC) Study. Am J Kidney Dis 55:31-41.

33. Kronenberg F (2009) Emerging risk factors and markers of chronic kidney disease progression. Nat Rev Nephrol 5:677-89.

34. Pillebout E, Burtin M, Yuan HT, Briand P, Woolf AS, et al. (2001) Proliferation and remodeling of the peritubular microcirculation after nephron reduction: association with the progression of renal lesions. Am J Pathol 159:547-60.

35. Viau A, El Karoui K, Laouari D, Burtin M, Nguyen C, et al. (2010) Lipocalin 2 is essential for chronic kidney disease progression in mice and humans. J Clin Invest 120:4065-76.

36. Schmid H, Boucherot A, Yasuda Y, Henger A, Brunner B, et al. (2006) Modular activation of nuclear factor-kappaB transcriptional programs in human diabetic nephropathy. Diabetes 55:2993-3003.

37. Al-Aly Z, Zeringue A, Fu J, Rauchman MI, McDonald JR, et al. (2010) Rate of Kidney Function Decline Associates with Mortality. J Am Soc Nephrol 21:1961-9.

38. Dalrymple LS, Katz R, Kestenbaum B, Shlipak MG, Sarnak MJ, et al. (2011) Chronic Kidney Disease and the Risk of End-Stage Renal Disease versus Death. J Gen Intern Med 26:379-85.

39. Agarwal R, Bunaye Z, Bekele DM, Light RP (2008) Competing risk factor analysis of end-stage renal disease and mortality in chronic kidney disease. Am J Nephrol 28:569-75.

40. Borthwick E, Ferguson A Perioperative acute kidney injury: risk factors, recognition, management, and outcomes. BMJ 341:c3365.

41. Kelly KJ, Dominguez JH Rapid Progression of Diabetic Nephropathy Is Linked to Inflammation and Episodes of Acute Renal Failure. Am J Nephrol 32:469-75.

42. van Kuijk JP, Flu WJ, Chonchol M, Hoeks SE, Winkel TA, et al. (2010) Temporary perioperative decline of renal function is an independent predictor for chronic kidney disease. Clin J Am Soc Nephrol 5:1198-204.

43. James MT, Ghali WA, Tonelli M, Faris P, Knudtson ML, et al. Acute kidney injury following coronary angiography is associated with a long-term decline in kidney function. Kidney Int 78:803-9.

44. Ronco C, McCullough PA, Anker SD, Anand I, Aspromonte N, et al. Cardiorenal syndromes: an executive summary from the consensus conference of the Acute Dialysis Quality Initiative (ADQI). Contrib Nephrol 165:54-67.

45. Winkelmayer WC, Owen WF, Jr., Levin R, Avorn J (2003) A propensity analysis of late versus early nephrologist referral and mortality on dialysis. J Am Soc Nephrol 14:486-92.

46. Ward MM (2009) Access to care and the incidence of end-stage renal disease due to diabetes. Diabetes Care 32:1032-6.

47. Soderland P, Lovekar S, Weiner DE, Brooks DR, Kaufman JS Chronic kidney disease associated with environmental toxins and exposures. Adv Chronic Kidney Dis 17:254-64.

48. Mann JF, Schmieder RE, McQueen M, Dyal L, Schumacher H, et al. (2008) Renal outcomes with telmisartan, ramipril, or both, in people at high vascular risk (the ONTARGET study): a multicentre, randomised, double-blind, controlled trial. Lancet 372:547-53.

49. Levey AS, Coresh J, Greene T, Stevens LA, Zhang YL, et al. (2006) Using standardized serum creatinine values in the modification of diet in renal disease study equation for estimating glomerular filtration rate. Ann Intern Med 145:247-54.

50. Willer CJ, Li Y, Abecasis GR METAL: fast and efficient meta-analysis of genomewide association scans. Bioinformatics 26:2190-1.

51. DerSimonian R, Laird N (1986) Meta-analysis in clinical trials. Control Clin Trials 7:177-88.

52. Gauderman WJ (2002) Sample size requirements for matched case-control studies of gene-environment interaction. Stat Med 21:35-50.

53. Johnson AD, Handsaker RE, Pulit SL, Nizzari MM, O'Donnell CJ, et al. (2008) SNAP: a web-based tool for identification and annotation of proxy SNPs using HapMap. Bioinformatics 24:2938-9.

54. Dixon AL, Liang L, Moffatt MF, Chen W, Heath S, et al. (2007) A genome-wide association study of global gene expression. Nat Genet 39:1202-7.

55. Göring HH, Curran JE, Johnson MP, Dyer TD, Charlesworth J, et al. (2007) Discovery of expression QTLs using large-scale transcriptional profiling in human lymphocytes. Nat Genet 39:1208-16.

56. Schadt EE, Molony C, Chudin E, Hao K, Yang X, et al. (2008) Mapping the genetic architecture of gene expression in human liver. PLoS Biol 6:e107.

57. (1989) The Atherosclerosis Risk in Communities (ARIC) Study: design and objectives. The ARIC investigators. Am J Epidemiol 129:687-702.

58. Fried LP, Borhani NO, Enright P, Furberg CD, Gardin JM, et al. (1991) The Cardiovascular Health Study: design and rationale. Ann Epidemiol 1:263-76.

59. Firmann M, Mayor V, Vidal PM, Bochud M, Pecoud A, et al. (2008) The CoLaus study: a population-based study to investigate the epidemiology and genetic determinants of cardiovascular risk factors and metabolic syndrome. BMC Cardiovasc Disord 8:6.

60. Dawber TR, Kannel WB, Lyell LP (1963) An approach to longitudinal studies in a community: the Framingham Study. Ann N Y Acad Sci 107:539-56.

61. Feinleib M, Kannel WB, Garrison RJ, McNamara PM, Castelli WP (1975) The Framingham Offspring Study. Design and preliminary data. Prev Med 4:518-25.

62. Garrison RJ, Castelli WP, Feinleib M, Kannel WB, Havlik RJ, et al. (1979) The association of total cholesterol, triglycerides and plasma lipoprotein cholesterol levels in first degree relatives and spouse pairs. Am J Epidemiol 110:313-21.

63. Splansky GL, Corey D, Yang Q, Atwood LD, Cupples LA, et al. (2007) The Third Generation Cohort of the National Heart, Lung, and Blood Institute's Framingham Heart Study: design, recruitment, and initial examination. Am J Epidemiol 165:1328-35.

64. Wichmann HE, Gieger C, Illig T (2005) KORA-gen--resource for population genetics, controls and a broad spectrum of disease phenotypes. Gesundheitswesen 67 Suppl 1:S26-30.

65. Hofman A, Grobbee DE, de Jong PT, van den Ouweland FA (1991) Determinants of disease and disability in the elderly: the Rotterdam Elderly Study. Eur J Epidemiol 7:403-22.

66. Hofman A, Breteler MM, van Duijn CM, Janssen HL, Krestin GP, et al. (2009) The Rotterdam Study: 2010 objectives and design update. Eur J Epidemiol 24:553-72.

67. Hofman A, Breteler MM, van Duijn CM, Krestin GP, Pols HA, et al. (2007) The Rotterdam Study: objectives and design update. Eur J Epidemiol 22:819-29.

68. Perrone RD, Madias NE, Levey AS (1992) Serum creatinine as an index of renal function: new insights into old concepts. Clin Chem 38:1933-53.

69. John U, Greiner B, Hensel E, Ludemann J, Piek M, et al. (2001) Study of Health In Pomerania (SHIP): a health examination survey in an east German region: objectives and design. Soz Praventivmed 46:186-94.

70. Volzke H, Alte D, Schmidt CO, Radke D, Lorbeer R, et al. Cohort Profile: The Study of Health in Pomerania. Int J Epidemiol.

71. Gutierrez OM, Mannstadt M, Isakova T, Rauh-Hain JA, Tamez H, et al. (2008) Fibroblast growth factor 23 and mortality among patients undergoing hemodialysis. N Engl J Med 359:584-92.

72. Friedman DJ, Afkarian M, Tamez H, Bhan I, Isakova T, et al. (2009) Klotho variants and chronic hemodialysis mortality. J Bone Miner Res 24:1847-55.

73. Longenecker JC, Coresh J, Powe NR, Levey AS, Fink NE, et al. (2002) Traditional cardiovascular disease risk factors in dialysis patients compared with the general population: the CHOICE Study. J Am Soc Nephrol 13:1918-27.

74. Kronenberg F, Kuen E, Ritz E, Junker R, Konig P, et al. (2000) Lipoprotein(a) serum concentrations and apolipoprotein(a) phenotypes in mild and moderate renal failure. J Am Soc Nephrol 11:105-15.

75. Boes E, Fliser D, Ritz E, Konig P, Lhotta K, et al. (2006) Apolipoprotein A-IV predicts progression of chronic kidney disease: the mild to moderate kidney disease study. J Am Soc Nephrol 17:528-36.

76. Gaspari F, Perico N, Matalone M, Signorini O, Azzollini N, et al. (1998) Precision of plasma clearance of iohexol for estimation of GFR in patients with renal disease. J Am Soc Nephrol 9:310-3.

77. Böger CA, Stubanus M, Haak T, Götz AK, Christ J, et al. (2007) Effect of MTHFR C677T genotype on survival in type 2 diabetes patients with end-stage diabetic nephropathy. Nephrol Dial Transplant 22:154-62.

78. Böger CA, Haak T, Götz AK, Christ J, Ruff E, et al. (2006) Effect of ACE and AT-2 inhibitors on mortality and progression to microalbuminuria in a nested case-control study of diabetic nephropathy in diabetes mellitus type 2: results from the GENDIAN study. Int J Clin Pharmacol Ther 44:364-74.

79. Wanner C, Krane V, Marz W, Olschewski M, Mann JF, et al. (2005) Atorvastatin in patients with type 2 diabetes mellitus undergoing hemodialysis. N Engl J Med 353:238-48.

80. Heid IM, Wagner SA, Gohlke H, Iglseder B, Mueller JC, et al. (2006) Genetic architecture of the APM1 gene and its influence on adiponectin plasma levels and parameters of the metabolic syndrome in 1,727 healthy Caucasians. Diabetes 55:375-84.
